# Machine learning and deep learning for diagnosis of endometriosis: systematic review and meta-analysis

**DOI:** 10.3389/fmed.2026.1907483

**Published:** 2026-10-15

**Authors:** Jiayi Guo, Xue Liu, Huiying Zhang, Shaobin Wei

**Affiliations:** 1 Chengdu University of Traditional Chinese Medicine, Chengdu, China; 2 Department of Gynecology, Hospital of Chengdu University of Traditional Chinese Medicine, Chengdu, China

**Keywords:** deep learning, diagnosis, diagnostic accuracy, endometriosis, machine learning, meta-analysis

## Abstract

**Objective:**

To assess the diagnostic accuracy of machine learning (ML) and deep learning (DL) models for endometriosis.

**Methods:**

A systematic review and meta-analysis were conducted in accordance with PRISMA guidelines (PROSPERO: CRD420251231469). Databases were searched through November 24, 2025. Twenty-two eligible studies were included. Risk of bias was assessed using QUADAS-2. Pooled analysis was conducted in Stata 18.0.

**Results:**

Pooled sensitivity was 0.90 (95% CI 0.86–0.93), specificity 0.75 (95% CI 0.64–0.84), and SROC-AUC 0.92 (95% CI 0.89–0.94). A positive prediction increased disease probability from 10% to 29%, whereas a negative prediction reduced it to 1%. Multicenter design and DL were associated with lower sensitivity. Results were robust.

**Conclusions:**

ML/DL models demonstrate high diagnostic performance and are promising for non-invasive auxiliary diagnosis. Large multicenter prospective studies and multimodal models are needed to enable clinical translation.

**Systematic Review Registration**: https://www.crd.york.ac.uk/PROSPERO/view/CRD420251231469, identifier CRD420251231469

## Background

Endometriosis is a chronic, estrogen-driven inflammatory pain disorder characterized by the presence of endometrium-like tissue outside the uterus, most commonly within the pelvic cavity, where it proliferates and invades adjacent tissues. Common clinical manifestations include dysmenorrhea, infertility, and chronic pelvic pain (1). The condition affects approximately 10% of women of reproductive age worldwide, with a substantially higher prevalence of 50% to 80% among women presenting with pelvic pain or infertility, and more than 176 million women are affected globally (1, 2). Beyond physical suffering, endometriosis significantly impairs mental health, work productivity, and social relationships, with many patients experiencing anxiety, depression, and a reduced sense of overall well-being. Furthermore, the disease often requires lifelong management and is associated with multiple comorbidities, resulting in significant healthcare resource consumption and socioeconomic burden (1).

Despite the high prevalence and substantial clinical burden of endometriosis, diagnosis remains a major challenge in clinical practice. Currently, laparoscopic surgery with pathological biopsy remains the “gold standard” for definitive diagnosis (3). However, this approach is invasive, carries surgical risks, and incurs considerable costs, making it unsuitable for early screening or routine clinical assessment. Non-invasive diagnostic methods, including transvaginal ultrasound, magnetic resonance imaging, and serum biomarkers such as CA-125, have been widely adopted in clinical practice, yet each has notable limitations. Ultrasound and MRI results are highly dependent on operator experience, and both modalities demonstrate suboptimal sensitivity and specificity for deep-infiltrating or atypical lesions (4, 5); serum biomarkers lack sufficient specificity and sensitivity and are susceptible to interference from other inflammatory diseases or malignant tumors (6–8).

A significant number of patients experience considerable delays before receiving a definitive diagnosis. This delay is attributable to nonspecific clinical symptoms, a broad differential diagnosis, limited awareness among healthcare professionals, unnecessary investigations, and a lack of reliable noninvasive diagnostic tools. According to available research, the diagnosis of endometriosis is delayed by a period ranging from seven to twelve years globally (9, 10). These delays increase the risk of infertility, chronic discomfort, and surgical complications by allowing the condition to worsen. Accordingly, there is an urgent clinical need to develop accurate, non-invasive, and efficient diagnostic tools to shorten diagnostic delays, improve early detection rates, and optimize the management of endometriosis, a pressing priority in gynecological clinical practice.

In recent years, artificial intelligence (AI) technologies, particularly machine learning (ML) and deep learning (DL), have seen rapid growth in healthcare applications, demonstrating substantial potential for disease diagnosis, treatment, and higher-level analysis of large biomedical datasets (11, 12). Machine learning encompasses a diverse range of algorithms that can learn patterns from labeled or unlabeled data, enabling disease prediction and classification (13); Deep learning, a branch of machine learning, is especially well-suited for image analysis and intricate pattern recognition tasks because it uses deep neural networks, such as convolutional neural networks and 3D DenseNets, to automatically extract high-level features from raw data (14). In medical image diagnosis, DL models have achieved diagnostic performance comparable to or even surpassing that of human experts, and have made breakthrough progress in skin cancer classification, diabetic retinopathy screening, and chest x-ray-based pneumonia detection (15, 16). Given the limitations of conventional diagnostic approaches, researchers have begun exploring the application of AI to the early, noninvasive diagnosis of endometriosis. For instance, models based on CNNs that are trained on sufficiently large datasets have demonstrated a substantial enhancement in the accuracy of non-invasive endometriosis diagnosis (17). The model showed an area under the curve (AUC) of 0.981 and achieved an overall diagnostic accuracy of approximately 94%, according to a study employing radiomics to distinguish ovarian endometriomas from dermoid cysts (18). Another study evaluated multiple machine learning algorithms trained on clinical history and physical examination data from women with chronic pelvic pain but no radiologic abnormalities. The best-performing model achieved a sensitivity of 79.3% and a specificity of 74.2% in predicting superficial peritoneal endometriosis (SPE) (19).

Although the studies above indicate that ML and DL hold considerable promise for diagnosing endometriosis, the current body of evidence remains subject to important limitations. To date, no systematic understanding of the predictive accuracy of either ML or DL in this setting has been established. This study therefore aimed to determine the diagnostic accuracy of machine learning approaches—encompassing both conventional and deep learning models—for detecting endometriosis and to provide evidence-based recommendations to advance artificial intelligence in this field.

## Methods

### Study design and registration

This study followed the PRISMA guidelines to ensure quality and transparency. We also registered and approved the study protocol with the International Prospective Register of Systematic Reviews (PROSPERO) before starting the research, with registration ID CRD420251231469.

### Search strategy

This meta-analysis included studies that developed ML or DL models for diagnosing or predicting endometriosis. From the beginning through November 24, 2025, a comprehensive literature search was conducted in PubMed, Web of Science, Embase, SCOPUS, and the Cochrane Library. There were no restrictions on publication location or year, and both free-text keywords and MeSH (Medical Subject Headings) terms were used. Additionally, the Supplementary Materials include comprehensive search strategies for each database.

### Inclusion and exclusion criteria

To ensure the accuracy and reliability of the analysis, studies meeting the following inclusion criteria were selected for this meta-analysis: (1) Studies developing prediction models for endometriosis, with model performance evaluated using metrics including the area under the curve (AUC); (2) Original peer-reviewed research articles, including prospective cohort studies, retrospective cohort studies, case-control studies, and other comparable study designs; (3) Studies in which the primary predictive outcome was the diagnosis of endometriosis; (4) Articles published in English. Exclusion criteria were as follows: (1) Studies based on overlapping patient populations; (2) Meta-analyses, systematic reviews, single-arm trials, case reports, and expert opinion articles; (3) Studies for which full-text articles were unavailable; (4) Studies that did not employ machine learning methods, or those that used only simple logistic or linear regression models.

### Study selection and data extraction

All identified studies were imported into Zotero, and duplicate records were removed. Two reviewers separately checked abstracts and titles for eligibility. Full texts of potentially eligible studies were then evaluated, and the same two reviewers independently extracted relevant data. Any disagreements during abstract or full-text screening were resolved by consulting a third independent reviewer, who made the final decision. The first author's name, publication year, country, study design, model category, sample size, and prediction results (accuracy, sensitivity, specificity, F1-score, and AUC) were among the information extracted from the included studies.

For studies that reported multiple algorithms, we selected the best-performing model based on the area under the curve (AUC) to standardize the inclusion process and ensure that the summary estimates reflected the optimal achievable performance for each data modality. When results from multiple datasets (e.g., training, validation, and test sets) were presented, we preferentially extracted metrics from the independent test set (i.e., the hold-out set not used for model training or hyperparameter tuning); if no dedicated test set was used, the cross-validation estimates were adopted. For studies that presented multiple diagnostic thresholds, we prioritized the threshold reported as the primary or recommended clinical cut-off by the original authors; if none was specified, we selected the threshold corresponding to the Youden index to maintain objectivity. This hierarchical approach ensured that each study contributed a single, non-overlapping set of effect estimates to the meta-analysis.

### Risk of bias assessment

The QUADAS-2 (Quality Assessment of Diagnostic Accuracy Studies 2) tool was used to evaluate the applicability and risk of bias of the included studies (20). This tool assesses four domains: patient selection, index test, reference standard, and flow and timing. Each domain contains specific signaling questions answered as “yes,” “no,” or “unclear,” corresponding to low, high, or unclear risk of bias, respectively. A study was considered to have a low risk of bias if all key questions across all domains were answered “yes.” A “no” response indicated potential bias, and assessors judged the level of bias in accordance with established guidelines. An “unclear” rating indicated insufficient information to make a definitive determination.

### Statistical analysis

Stata 18.0 was used for all statistical analyses. Fourfold table data were extracted from the included studies; when the original studies did not directly report diagnostic 2 × 2 tables, these were calculated from reported sensitivity, specificity, accuracy, and total sample size. Pooled estimates of sensitivity, specificity, positive likelihood ratio (PLR), negative likelihood ratio (NLR), and diagnostic odds ratio (DOR) were computed. A summary receiver operating characteristic (SROC) curve was created by pooling sensitivity and specificity concurrently, taking their correlation into account, using a bivariate random-effects model. The AUC of the SROC curve was calculated to assess overall diagnostic performance. The *I*^2^ statistic and Cochran's *Q*-test were employed to assess heterogeneity among studies; an *I*^2^ value exceeding 50% was considered indicative of substantial heterogeneity. Multivariable random-effects meta-regression was used to account for confounding variables and identify covariates independently associated with diagnostic performance, while subgroup analyses were conducted to investigate potential sources of heterogeneity. Regression coefficients were estimated using restricted maximum-likelihood estimation, with the Knapp–Hartung adjustment applied for small-sample correction. Influential and outlying studies were identified using bivariate boxplots, and leave-one-out analyses were performed to assess the robustness of the pooled estimates. Publication bias was evaluated using Deeks' funnel plot asymmetry test, with *P* < 0.10 considered indicative of significant publication bias. If the trim-and-fill method failed to converge, a sensitivity analysis excluding studies with the lowest precision was performed as an alternative. All statistical tests were two-sided, with *P* < 0.05 considered statistically significant.

## Results

### Study selection

The study screening flowchart is shown in Figure 1. Initially, 2050 studies were identified through database searches. After an in-depth evaluation of 59 full-text studies for eligibility, 22 studies were ultimately selected for the final analysis.

**Figure 1:**
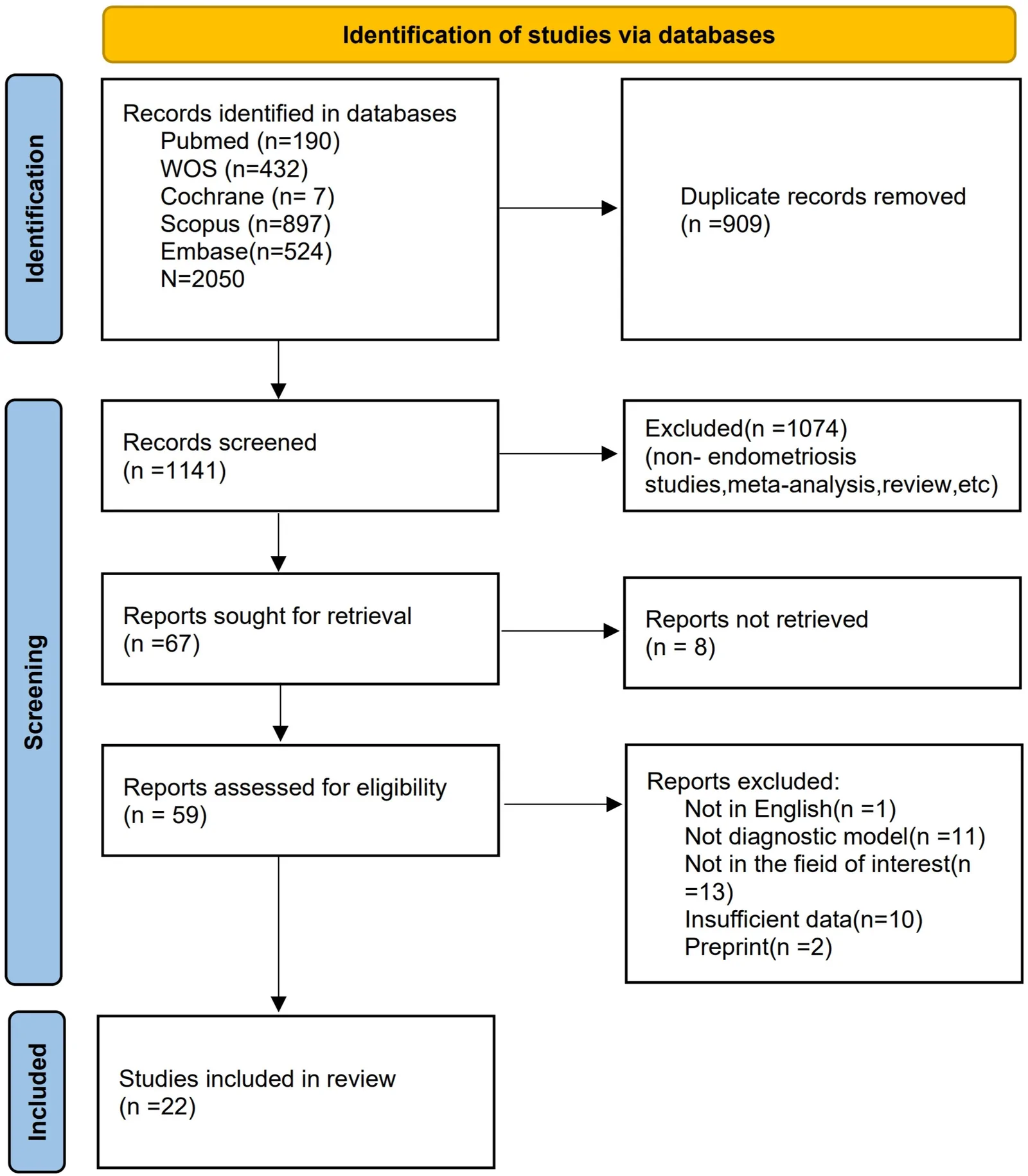
Flowchart of study selection process.

### Study characteristics

Studies were published between 2021 and 2025 and spanned 12 countries across Asia, Europe, the Americas, and Oceania, including China, Australia, Brazil, France, Germany, India, Iran, Israel, Italy, Spain, Thailand, and the United States. Regarding study design, 17 were retrospective, 3 were prospective, 1 was a case–control study, and 1 was a cross-sectional study. Single-center studies predominated (*n* = 15), with 7 multicenter studies also included. In terms of model type, 16 studies employed ML models and 6 employed DL models. Regarding validation strategies, 15 studies used internal validation only (cross-validation or hold-out), 7 employed multi-center designs providing implicit external validation, and no study performed independent external or temporal validation (Table 1).

**Table 1:** Characteristics of included studies.

Authors	Country	Research type	Patient source	Sample size	Data type	Disease subtype	Validation method	Model classification	Sensitivity	Specificity	AUC	Accuracy	Precision	F1
Martins MS et al. (21)	Brazil	Prospective	Single center	100 (50 endometriosis-positive, 50 endometriosis-negative)	Urine ATR-FTIR spectra	Not specified	Internal	ML	0.93	0.57	0.77	0.76	0.70	0.80
Enamorado-Díaz E et al. (22)	Spain	Retrospective	Single center	5,143 (270 endometriosis-positive, 4,873 endometriosis-negative)	Clinical EHR data; exome genetic data	No subtype stratification	Internal	ML	0.9,576	0.2,245	0.9,566	0.9,566	0.9,441	0.9,477
Moassefi M et al. (23)	USA	Retrospective	Single center	751 (395 endometriosis cases, 356 controls)	Multi-sequence MRI	No subtype stratification	Internal	ML	0.976	0.720	0.911	0.8,549	0.7,942	0.881
Soo Z et al. (24)	Australia	Retrospective	Multicenter	1,071	Genomic data	No subtype stratification	External	DL	0.6,884	0.6,077	0.6,729	0.6,739	0.6,738	0.6,809
Zhao N et al. (25)	China	Retrospective	Single center	309 (endometriosis cases 106, 203 controls)	Serological indicators	Ovarian endometriosis	Internal	ML	0.8,621	0.7,414	0.85	0.7,816	0.625	0.7,246
Guerriero S et al. (26)	Italy	Prospective	Multicenter	Training set: 222 Test set: 110	Ultrasound images	Rectosigmoid deep endometriosis	Internal	ML	0.72	0.73	0.8,173	0.73	0.5,227	0.6,053
Visalaxi et al. (27)	India	Retrospective	Single center	Training: 3,600 Validation: 1,200 Test set:1,200	Laparoscopic images	No subtype stratification	Internal	DL	0.82	0.72	0.78	0.9	0.83	0.82
Zaidi SA et al. (28)	Thailand	Retrospective	Single center	Training set: 7,000 Validation:2,000 Test set: 1,000	Laparoscopic images	No subtype stratification	Internal	DL	0.94	0.9,183	0.92	0.935	0.9,307	0.9,353
Xu J et al. (29)	China	Retrospective	Single center	147	Ultrasound images	Deep infiltrating endometriosis	Internal	DL	0.8,871	0.8,696	0.88	0.8,843	0.9,735	0.9,397
Tore U et al. (30)	Spain	Retrospective	Multicenter	controls 121,527, endometriosis cases 1,029	Electronic medical records	No subtype stratification	Internal	ML	0.686	0.629	0.725	0.6,297	0.015	0.0,293
Yang M et al. (31)	China	Retrospective	Single center	118endometriosis cases, controls 206	Transvaginal ultrasound + MRI images	Deep pelvic endometriosis	Internal	DL	0.9,545	0.917	Not mentioned	0.9,237	0.9,899	0.954
Kupec T et al. (32)	Germany	Retrospective	Single center	36 endometriosis cases, 16 controls	Serum circulating miRNAs	No subtype stratification	Internal	ML	0.714	0.625	0.76	0.714	0.8	0.632
Miao K et al. (33)	China	Retrospective	Single center	132 endometriosis cases,52 controls	Ultrasound images	Ovarian endometriosis	Internal	ML	0.9	0.9	0.9	0.9	0.96	0.9,278
Kasoha M et al. (34)	Germany	Case-Control Study	Single center	59endometriosis, 28 controls	Serum inflammatory markers	No subtype stratification	External	ML	0.925	0.583	0.7,948	0.818	0.831	0.8,761
Schoeman EM et al. (35)	Australia	Retrospective	Multicenter	89 endometriosis cases, 130 controls	Plasma protein biomarkers	No subtype stratification	External	ML	0.98	0.96	0.997	0.97	0.95	0.965
Sofiane Bendifallah S et al. (36)	France	Retrospective	Multicenter	Training set: 1,734 Validation: 100	Patient-reported clinical and symptom features	No subtype stratification	External	ML	0.93	0.92	0.93	0.93	0.91	0.92
Nouri B et al. (37)	Iran	Retrospective	Single center	Training set: 378 Test set: 127	Ultrasound images	No subtype stratification	Internal	ML	0.93	0.92	0.93	0.93	0.91	0.92
Li Y et al. (38)	China	Retrospective	Single center	100 (61 endometriosis, 39 controls)	Ultrasound images	Ovarian endometriosis	Internal	DL	0.9,344	0.7,948	Not mentioned	0.88	0.8,769	0.9,047
Konrad L et al. (39)	Germany	Prospective	Single center	269 (177 endometriosis, 92controls)	Clinical features	No subtype stratification	Internal	ML	0.904	0.75	Not mentioned	0.8,513	0.874	0.889
Xie R et al. (40)	China	Retrospective	Multicenter	Training set: 204 Validation: 96	Gene expression data	No subtype stratification	External	ML	0.719	0.719	0.769	0.719	0.772	0.732
Goldstein A et al. (41)	Israel	cross-sectional study	Multicenter	886 (474 endometriosis, 412 controls)	Self-reported symptom questionnaire	No subtype stratification	Internal	ML	0.932	0.946	0.939	0.939	0.954	0.943
Snyder DL et al. (42)	USA	Retrospective	Single center	778 (654 endometriosis, 134 controls)	Clinical features	No subtype stratification	Internal	ML	0.962	0.111	0.761	0.816	0.84	0.897

### Methodological quality

For the QUADAS-2 risk of bias assessment (Figure 2), we identified two studies with a high risk of bias (34, 41), three with an unclear risk of bias (22, 29, 39), and 17 with an overall low risk. Among these, one (34) was a case-control study, and another (41) involved two gold standards, resulting in a high risk of selection bias. Furthermore, three studies did not specify the interval between the index test and the gold standard, resulting in an unclear risk of bias. We used RevMan 5.4 to generate the quality assessment plot.

**Figure 2:**
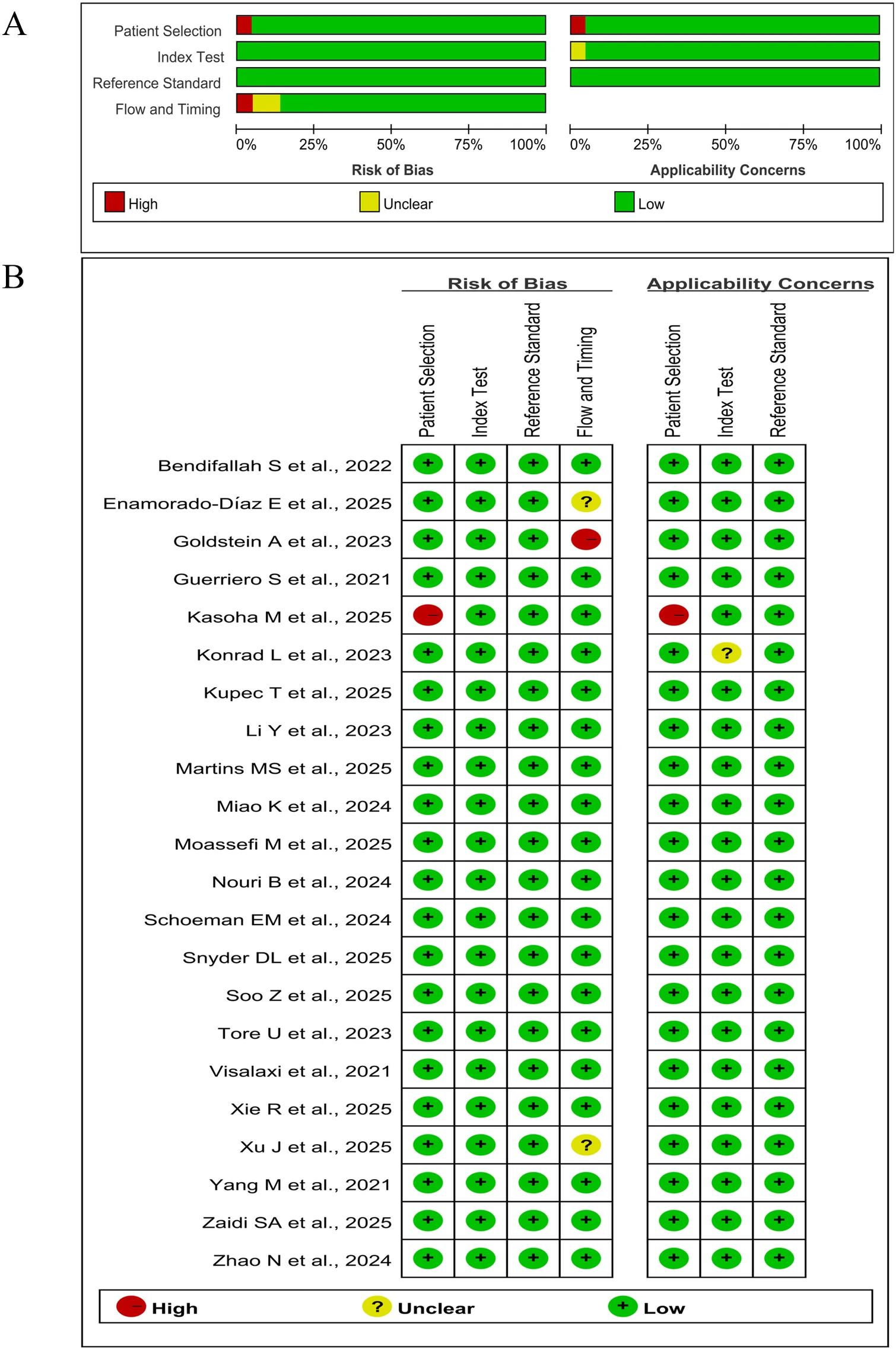
Risk of bias **(A)** graph and **(B)** summary.

### Performance of ML and DL models in predicting endometriosis

A random-effects model was employed to estimate the pooled diagnostic parameters. Forest plots for the AUC, sensitivity, specificity, PLR, NLR, DOR, and SROC were incorporated into this meta-analysis. Based on the diagnostic meta-analysis results, the pooled sensitivity and specificity across the 22 included studies were 0.90 (95% CI 0.86–0.93) and 0.75 (95% CI 0.64–0.84), respectively (Figure 3). In addition, the overall diagnostic accuracy of ML and DL models in predicting endometriosis was evaluated. The Spearman correlation coefficient was statistically significant (*P* < 0.01) in the pooled analysis. The chi-square test yielded a *P* value less than 0.05, indicating significant heterogeneity among the included studies. Additionally, the *I*^2^ statistics for sensitivity and specificity were recorded at 99.00% and 99.79%, respectively. The median results of the pooled predictive data are as follows: the pooled PLR was 3.65 (95% CI 2.44–5.46) (Figure 4A), the pooled NLR was 0.13 (95% CI 0.08–0.20) (Figure 4B), and the pooled DOR was 27.57 (95% CI 13.82–54.98) (Figure 4C). The corresponding SROC curve is presented in Figure 5. ML or DL models for endometriosis prediction have strong diagnostic value, as evidenced by an overall SROC area under the curve of 0.92 (95% CI 0.89–0.94).

**Figure 3:**
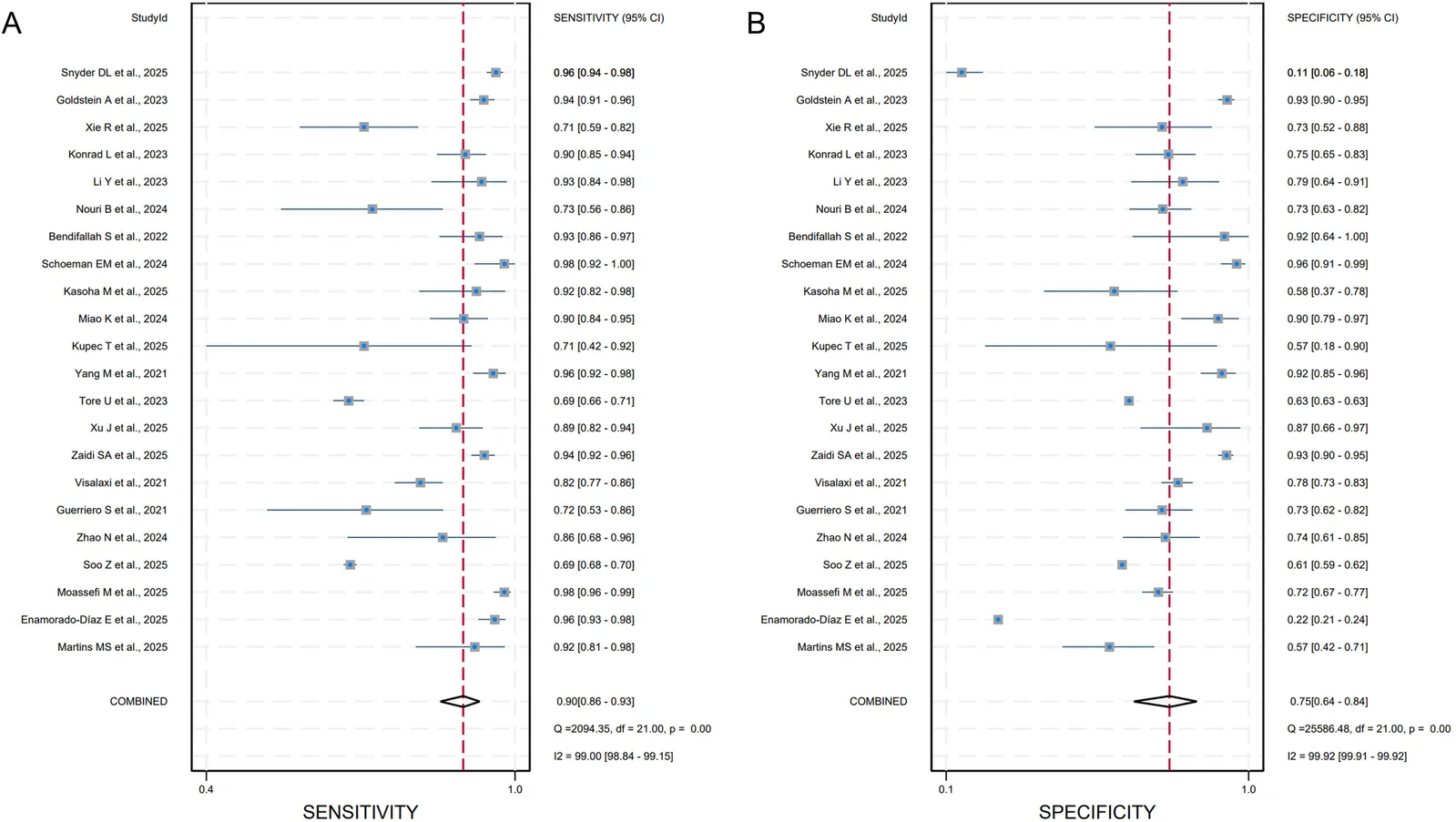
Forest plots in sensitivity and specificity of machine learning (ML) or deep learning (DL) models.

**Figure 4:**
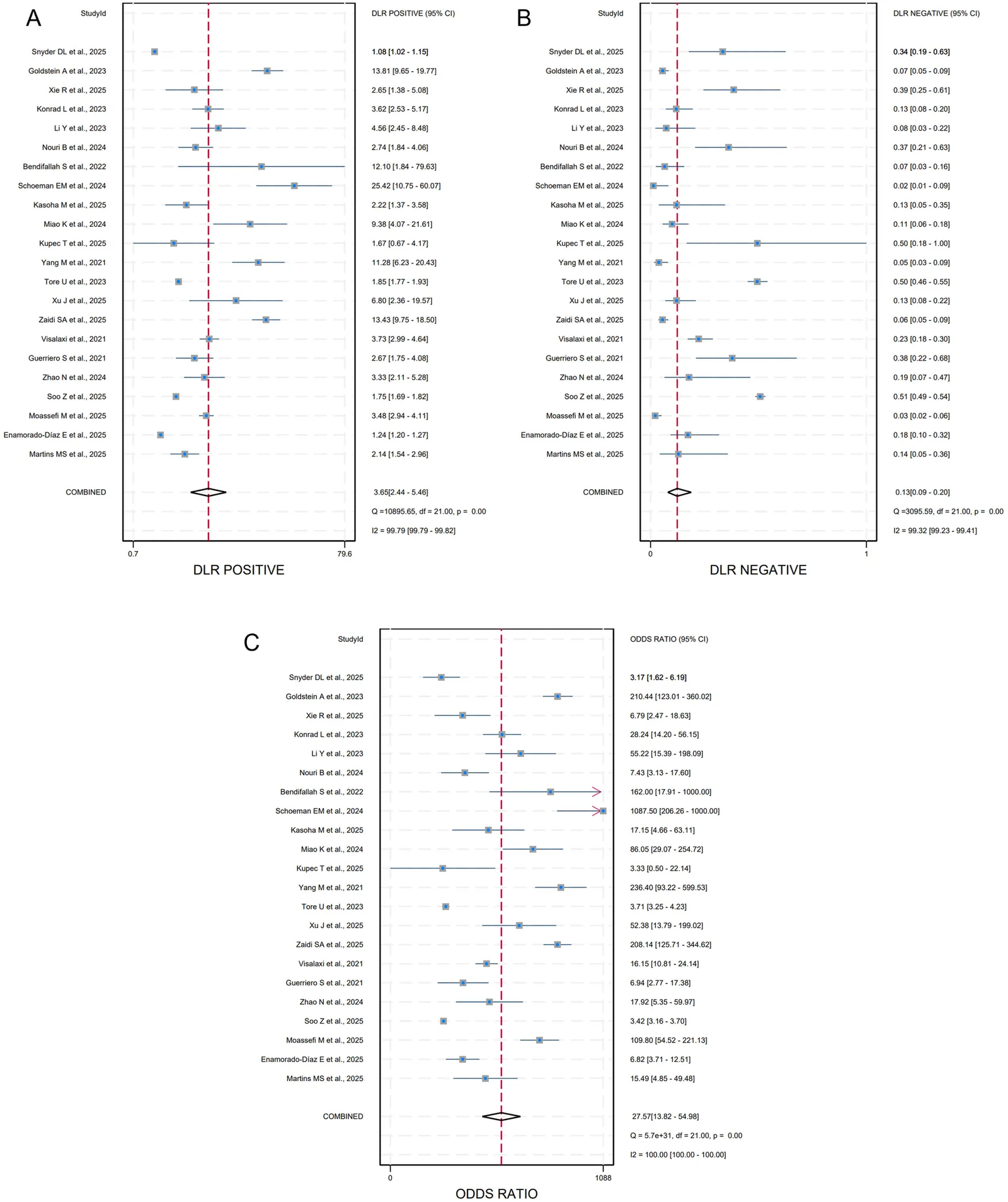
Comprehensive performance of machine learning (ML) or deep learning (DL) in the prediction of endometriosis. Forest plots of PLR **(A)**, NLR **(B)**, and DOR **(C)**.

**Figure 5:**
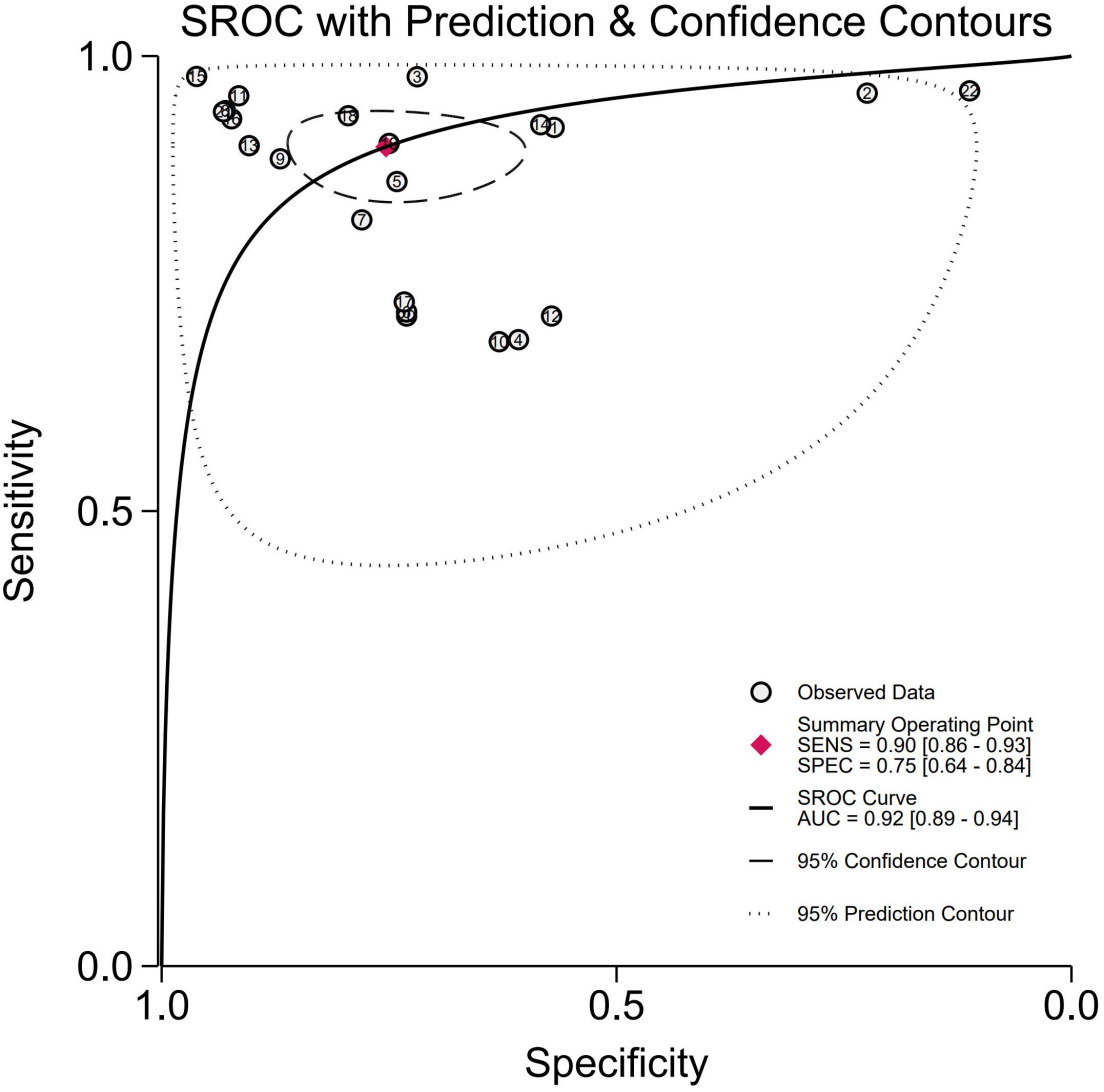
The SROC graph for the studies.

### Clinical value analysis

The clinical utility of machine learning methods for predicting endometriosis was evaluated using pretest and posttest probability plots. Based on a literature review, the morbidity of endometriosis among reproductive-age women was reported to be 10% (2); therefore, the pretest probability was set at 10%. When the model predicted a positive result, the probability increased from 10% to 29%, whereas a negative result reduced it to 1% (Figure 6).

**Figure 6:**
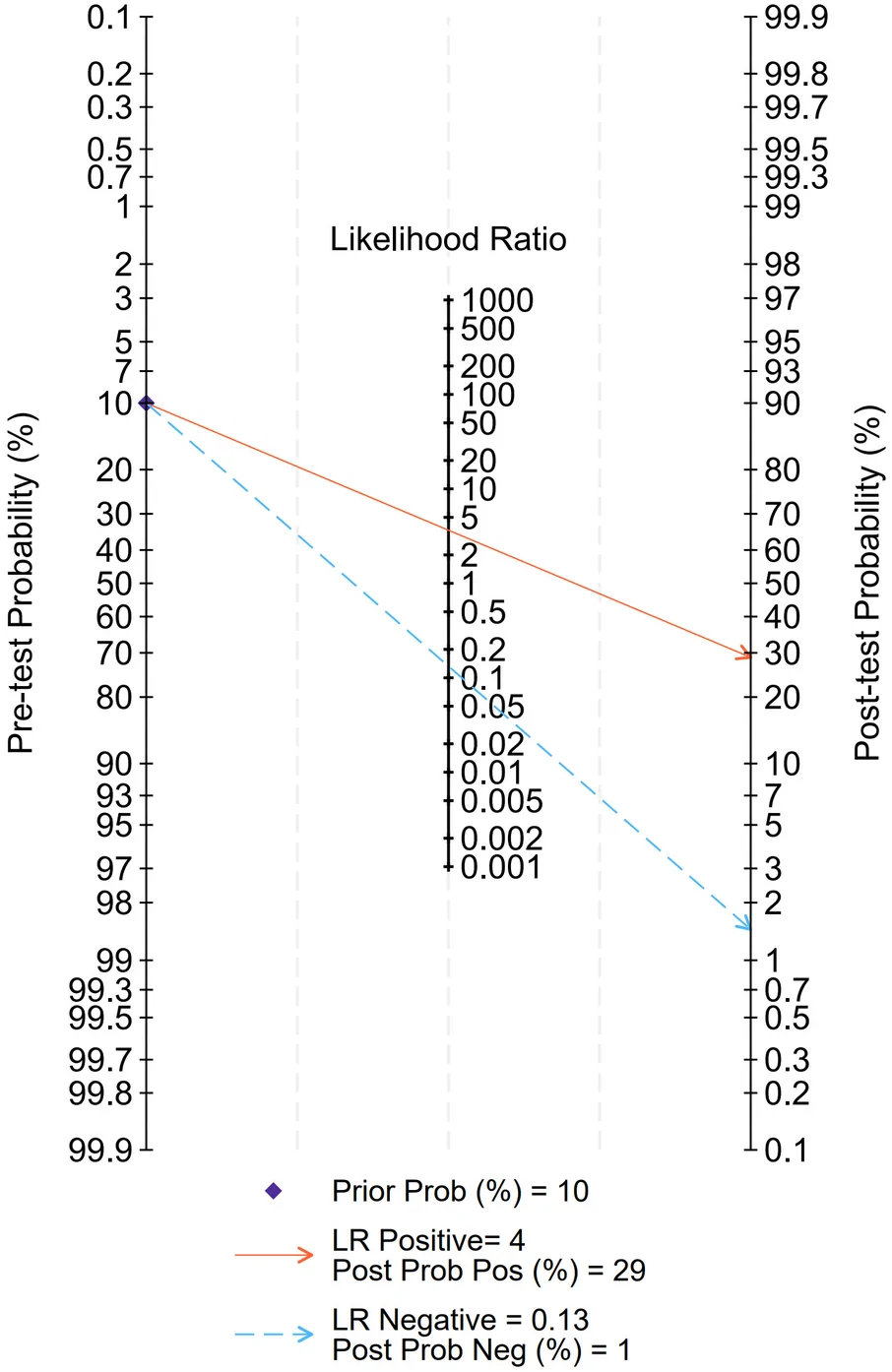
Fagan nomogram of traditional machine learning (ML) or deep learning (DL) models for diagnosing endometriosis. This nomogram shows the pretest probability in the first column, the likelihood ratio in the second, and the posttest probability in the third.

### Subgroup analysis

To explore potential sources of heterogeneity, subgroup analyses were performed according to the number of study centers, diagnostic model type, sample size, national economic level, publication year, validation method, and data type. The results are summarized in Table 2. Univariate analyses indicated that the number of study centers, model type, sample size, national economic level, validation method, and publication year were significantly associated with sensitivity. In contrast, specificity was mainly associated with publication year and data type. To control for confounding factors and identify independent influencing factors, we further performed multivariate meta-regression.

**Table 2:** Subgroup analysis of diagnostic accuracy.

Subgroup	Category	No. of studies	Sensitivity (95% CI)	*p*-value (Sensitivity)	Specificity (95% CI)	*p*-value (Sensitivity)
Centers	Multicenter	7	0.85 (0.76–0.93)	0.00	0.83 (0.70–0.96)	0.99
	Single-center	15	0.92 (0.89–0.95)		0.71 (0.58–0.84)	
Model	DL	6	0.89 (0.82–0.96)	0.01	0.84 (0.71–0.97)	0.91
	ML	16	0.90 (0.86–0.94)		0.71 (0.59–0.83)	
Sample size	Large (>200)	11	0.92 (0.89–0.96)	0.03	0.74 (0.60–0.88)	0.25
	Small (≤200)	11	0.87 (0.80–0.93)		0.77 (0.63–0.90)	
Country	Low/middle-income	11	0.89 (0.84–0.95)	0.00	0.84 (0.75–0.93)	0.66
	High-income	11	0.91 (0.86–0.95)		0.64 (0.49–0.79)	
Publication year	Recent (2025)	10	0.91 (0.86–0.96)	0.00	0.60 (0.44–0.77)	0.00
	Early (≤2024)	12	0.89 (0.84–0.94)		0.84 (0.76–0.93)	
Validation method	External	5	0.88 (0.79–0.97)	0.01	0.81 (0.64–0.99)	0.92
	Internal	17	0.90 (0.87–0.94)		0.74 (0.62–0.85)	
Data type	Imaging	9	0.915 (0.901–0.927	0.00	0.825 (0.805–0.844)	0.00
	Clinical/symptom	6	0.847 (0.833–0.861	0.00	0.614 (0.616–0.612)	0.00
	Omics/biomarker	7	0.698 (0.686–0.710)	0.00	0.617 (0.604–0.630)	0.00

### Meta-regression

We conducted a multivariate random-effects meta-regression analysis incorporating the six aforementioned covariates, with logit-transformed sensitivity and specificity as the outcome measures. This approach was employed to explore sources of heterogeneity between studies and to control for potential confounding factors. The findings are presented in Table 3. The overall test of the sensitivity model was statistically significant [F(6,15) = 4.81, *p* = 0.007], and the covariates jointly explained 78.2% of the between-study variance (adjusted R-squared = 78.2%), with residual heterogeneity minimal (*τ*^2^ = 0.0029, *I*^2^_res = 3.43%). After controlling for other factors, multicenter studies were significantly and negatively associated with logit sensitivity (coefficient = −0.167, 95% CI: −0.285 to −0.050, *p* = 0.008), indicating that the pooled sensitivity of multicenter studies was significantly lower than that of single-center studies. Similarly, the use of deep learning methods was independently associated with lower logit sensitivity (coefficient = −0.142, 95% CI: −0.268 to −0.015, *p* = 0.030), suggesting that studies employing DL methods may have lower sensitivity than those using non-DL methods. Sample size (*p* = 0.312), country (*p* = 0.094), validation method (*p* = 0.852),and publication year (*p* = 0.495) showed no statistically significant effects on sensitivity, indicating that the effects of publication year and country observed in the univariate subgroup analyses may be primarily confounded by study center type and method type.

**Table 3:** Multivariate meta-regression of factors associated with diagnostic accuracy.

Covariate	Logit-sensitivity		Logit-specificity	
	Coefficient (95% CI)	*P*-value	Coefficient (95% CI)	*P*-value
Centers	−0.167 (–0.285, –0.050)	**0** **.** **008** ^a^	0.307 (–0.142, 0.757)	0.166
Model	–0.142 (–0.268, –0.015)	**0**.**030**^a^	0.287 (–0.212, 0.787)	0.241
Sample size	0.073 (–0.076, 0.223)	0.312	–0.158 (–0.602, 0.287)	0.463
Country	0.121 (–0.023, 0.264)	0.094	0.282 (–0.223, 0.786)	0.254
Year	0.042 (–0.085, 0.169)	0.495	–0.356 (–0.776, 0.063)	0.091
Validation method	0.020 (−0.207, 0.248)	0.852	0.176 (−0.451, 0.803)	0.559
Constant	–0.168 (–0.357, 0.022)	0.080	–0.498 (–1.047, 0.051)	0.073

Coefficients represent change in logit-transformed sensitivity/specificity per unit change in covariate (binary covariates: 1 vs. 0). All models were fitted using restricted maximum likelihood (REML) with Knapp-Hartung modification. CI = confidence interval. Bold indicates statistical significance at *p* < 0.05.

a Bold values with superscript “a” indicate statistical significance at *p* < 0.05.

The overall test of the specificity model approached statistical significance [F(6,15) = 2.62, *p* = 0.065], with the covariates explaining 31.9% of the between-study variance (adjusted R-squared = 31.9%). However, residual heterogeneity remained high (*τ*^2^ = 0.139, *I*^2^_res = 93.8%), suggesting that unmeasured factors continue to influence specificity. Among the covariates, only publication year showed a marginally significant negative association (coefficient = −0.356, 95% CI: −0.776 to 0.063, *p* = 0.091), indicating a trend toward lower pooled specificity in more recent studies compared with earlier ones. The remaining covariates (centers, model, sample size, country, validation method) had no significant effect on specificity (all *p* > 0.15).

Collinearity diagnostics showed that the variance inflation factor (VIF) for all covariates was below 2, indicating no serious multicollinearity in the model.

In summary, the multivariable meta-regression indicated that multicenter studies and the use of deep learning methods were independently associated with lower sensitivity, whereas publication year had a near-significant negative effect on specificity, suggesting that specificity may have declined in more recent studies.

### Sensitivity analysis

To assess the robustness of the pooled results, we constructed a bivariate box plot (Figure 7) to identify potential outliers. Two studies fell outside the 95% confidence ellipse 22,42, suggesting they may be outliers.

**Figure 7:**
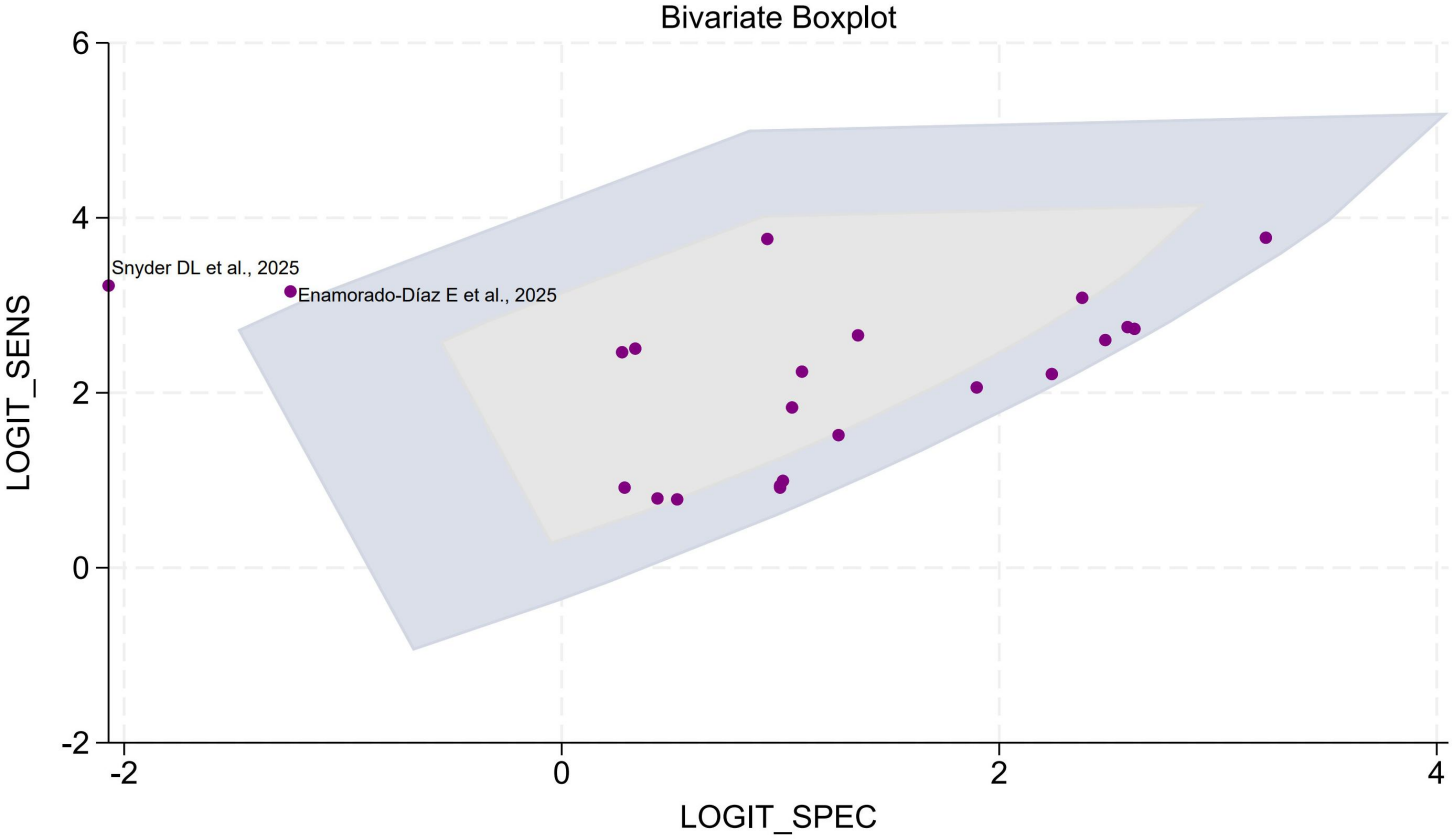
Bivariate boxplot of logit-transformed sensitivity and specificity. The ellipse represents the 95% confidence region for the joint distribution of the logit-sensitivity and logit-specificity. Studies located outside the ellipse (Snyder DL et al. (42) and Enamorado-Díaz E et al. (22) were identified as potential outliers.

To assess changes in pooled sensitivity and specificity, we then conducted a leave-one-out sensitivity analysis, progressively removing these two trials and refitting the bivariate random-effects model. The pooled original results (based on all 22 studies) and the results after exclusion are presented in Table 4. The confidence intervals for all re-estimated parameters showed substantial overlap with those from the original analysis, and none of the point estimates changed in a clinically meaningful way. These findings indicate that, although two studies were identified as graphical outliers, their exclusion did not substantially affect the pooled estimates of diagnostic accuracy. The overall results are therefore considered robust, suggesting that the aggregate estimates were not unduly influenced by individual outlier studies.

**Table 4:** Leave-one-out sensitivity analysis for the two potential outlier studies.

Analysis	Sensitivity (95% CI)	Specificity (95% CI)	Diagnostic odds Ratio (95% CI)
All studies	0.90 (0.86–0.93)	0.75 (0.64–0.84)	27.57 (13.82–54.98)
Excluding Snyder 2025	0.89 (0.85–0.93)	0.78 (0.69–0.85)	30.1 (15.0–60.2)
Excluding Enamorado-Díaz 2025	0.90 (0.85–0.93)	0.77 (0.68–0.85)	29.5 (14.5–60.0)

### Publication bias assessment

The Deeks funnel plot (Figure 8) showed an asymmetric distribution of study points, suggesting publication bias. An attempt was made to apply the trim-and-fill method to estimate the influence of missing studies; however, the algorithm failed to converge, likely due to the small number of included studies and the high degree of heterogeneity. As an alternative, a sensitivity analysis was conducted by excluding the five studies (29, 32, 34–36) with the lowest precision. After re-pooling, the DOR changed from the original 27.57 (95% CI: 13.82–54.98) to 21.68 (95% CI: 10.37–45.35), a limited change, with the confidence intervals overlapping those of the original estimate. Taken together with the results of the leave-one-out sensitivity analysis, the overall diagnostic accuracy estimate remains relatively robust despite the presence of publication bias.

**Figure 8:**
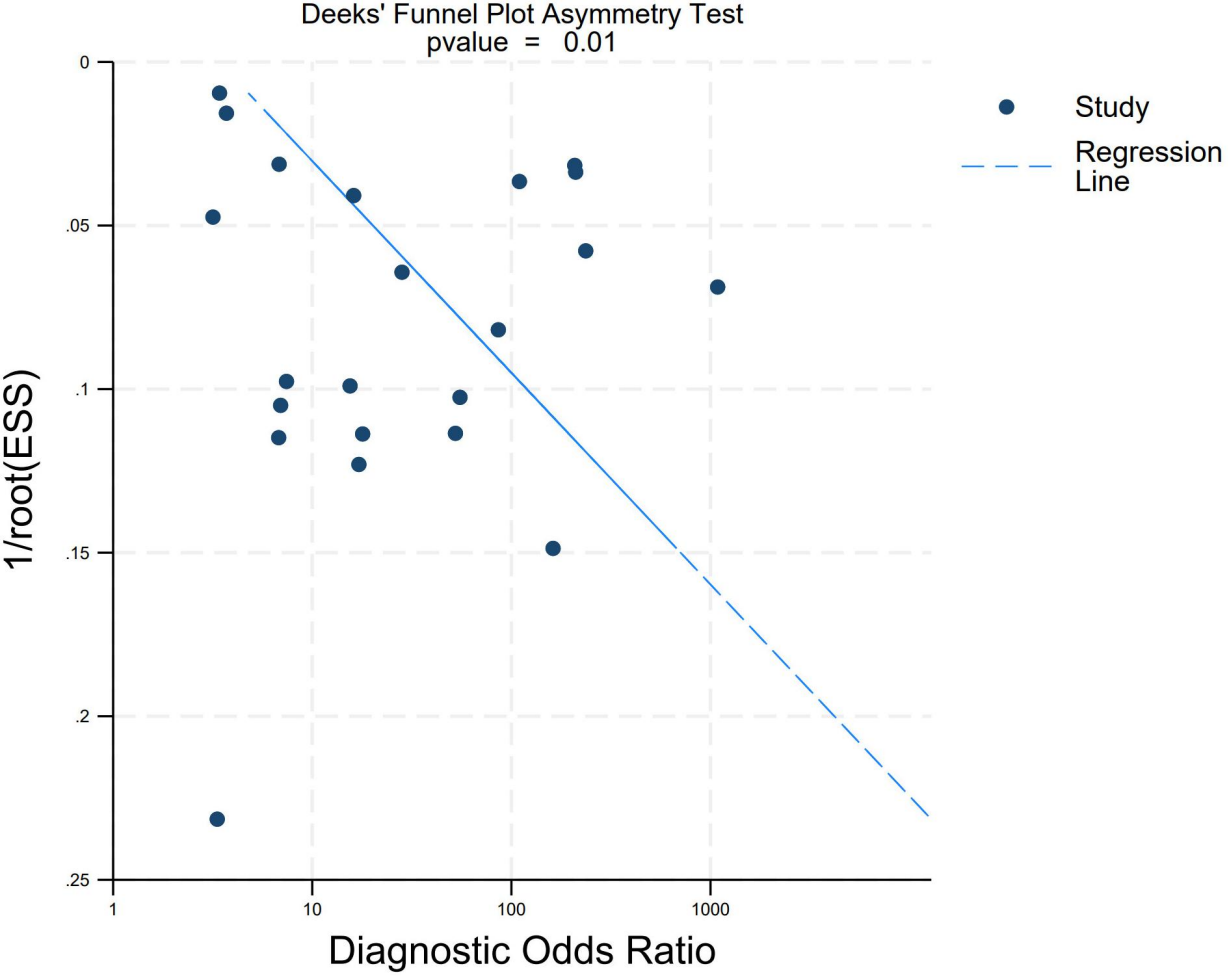
Funnel plot.

## Discussion

### Main findings

This meta-analysis synthesized evidence from 22 studies examining the diagnostic value of ML and DL models for endometriosis. The included studies spanned 12 countries and encompassed a variety of study designs and model types, providing the most comprehensive body of evidence to date in this field. A comprehensive evaluation of the pooled results showed that ML/DL models achieved a pooled sensitivity of 0.90 (95% CI: 0.86–0.93), a specificity of 0.75 (95% CI: 0.64–0.84), a diagnostic odds ratio (DOR) of 27.57 (95% CI: 13.82–54.98), and an area under the SROC curve of 0.92 (95% CI: 0.89–0.94) for the diagnosis of endometriosis. These findings indicate that ML/DL models demonstrate favorable overall diagnostic performance and hold considerable clinical value for the non-invasive diagnosis of endometriosis.

However, given that the included studies differ substantially in data modality (imaging, clinical variables, genomic data), target lesions, disease subtypes, and clinical settings, the pooled estimates should be interpreted as an exploratory synthesis rather than as a strictly validated clinical standard. We explicitly acknowledge that substantial heterogeneity was observed (*I*^2^ >99% for both sensitivity and specificity), which precludes a direct application of the summary estimates to clinical practice. The primary aim of pooling these heterogeneous data was not to establish a stringent diagnostic benchmark applicable across all settings, but rather to provide a broad, integrative overview of the current state of AI-driven diagnostic research—highlighting the overall potential and inherent limitations of ML/DL models in this field. Consequently, we emphasize that these models should currently be viewed as auxiliary aids to support clinical decision-making, rather than as replacements for existing diagnostic pathways.

Clinical value analysis, using a 10% prior probability of endometriosis in women of childbearing age as the baseline, showed that a positive model prediction raised the posterior probability to 29%, whereas a negative prediction reduced it to 1%. These findings indicate that ML/DL models can effectively increase the diagnostic probability of endometriosis and provide a high reference value for disease exclusion, thereby offering robust support for clinical diagnostic decision-making.

Subgroup analyses and multivariate meta-regression further identified key factors influencing model diagnostic performance. In the multivariate model, multicenter study design (coefficient = −0.167, *P* = 0.008) and the use of deep learning (DL) models (coefficient = −0.142, *P* = 0.030) were independently associated with reduced diagnostic sensitivity after adjusting for other covariates. In contrast, publication year showed a marginally significant negative effect on specificity (coefficient = −0.356, *P* = 0.091), suggesting a declining trend in model specificity among recently published studies. Notably, sample size, country income level, and validation method were significantly associated with sensitivity in univariate subgroup analyses but did not remain statistically significant in the multivariate model, indicating that their effects may be confounded by other study characteristics. The negative association between DL utilization and sensitivity should be interpreted with caution. It may not reflect an inherent algorithmic limitation of DL techniques but could stem from the predominant application of DL models to complex, high-dimensional imaging data, as well as variations in validation strategies and study designs across relevant subgroups. Although data type was not included in the multivariate model due to the limited number of studies, subgroup analysis showed that imaging-based studies achieved the highest pooled sensitivity and specificity, whereas omics/biomarker-based studies had the lowest, suggesting that data type may partly explain the observed differences.

Sensitivity analysis and publication bias assessment confirmed the robustness of the primary findings, with individual outlier studies and publication bias exerting no substantial impact on the overall conclusions.

### Comparison with other studies

Multiple non-invasive detection methods for endometriosis are under active investigation. In recent years, researchers have also explored additional non-invasive approaches. Circulating cell-free microRNAs (miRNAs) are an important source for biomarker discovery. Studies have found that the expression levels of miR-125b-5p, miR-150-5p, miR-342-3p, and miR-451a are significantly elevated in patients with endometriosis, while the levels of miR-3613-5p and let-7b are significantly reduced. When a classifier combining these miRNAs was validated in an independent subject group not included in the training set, an AUC is 0.94 (43). However, existing miRNA biomarker studies are predominantly small-scale exploratory investigations. Systematic reviews indicate that the overlap of differentially expressed miRNAs reported across studies is low, with poor reproducibility, inconsistent findings, and no unified diagnostic threshold standards. The vast majority of miRNA biomarker studies rely solely on internal validation or cross-validation, making it difficult to assess their performance in real-world clinical settings (44, 45). The rapid advancement of multi-omics technology has also provided new perspectives for the diagnosis of endometriosis. A panel of plasma protein biomarkers achieved an AUC of 0.997 for diagnosing severe endometriosis, while another study employing a multiple cytokine array for endometriosis diagnosis reported an area under the curve of 0.874 with a confidence interval of 0.81–0.94 (35, 46).

Although these detection methods have shown promise, they are all subject to technical bottlenecks and clinical application limitations. ML and DL technologies for the non-invasive diagnosis of endometriosis directly address the shortcomings of traditional biomarkers and single-detection methods. Compared with miRNA biomarker research, ML/DL models overcome the core challenges of high study heterogeneity, poor reproducibility, and insufficient validation. Although certain miRNA combination classifiers can achieve a high AUC of 0.94, they are constrained by small-sample exploratory study designs, with minimal overlap in differentially expressed miRNAs across studies, making replication in real-world clinical settings difficult. In contrast, ML/DL models can be trained and validated on large-sample data from multi-center, multi-type sources, integrating multi-dimensional data encompassing clinical history, imaging, and laboratory indices. By automatically mining latent associations among data through algorithms, these models effectively circumvent the subjectivity and limitations inherent in manual biomarker screening. Compared with multi-omics detection methods such as plasma protein and cytokine arrays, ML/DL models demonstrate a broader range of applicability and greater capacity for comprehensive analysis. Although plasma protein biomarkers can achieve high AUC values for severe endometriosis, their diagnostic efficacy for early-stage, mild, or atypical lesions has not been validated, indicating notable application scenario limitations. Cytokine arrays demonstrate relatively low diagnostic efficacy, and both approaches are limited to detecting and analyzing biomarkers along a single dimension, making it difficult to integrate the multi-source, heterogeneous diagnostic information encountered in clinical practice. ML/DL models, by contrast, are compatible with diverse data types, including multi-omics biomarkers, imaging data, and clinical features. By constructing multimodal fusion models, they enable comprehensive, integrated diagnosis of endometriosis, achieving high diagnostic accuracy across different disease stages and lesion types. Furthermore, through algorithmic optimization, ML/DL models can dynamically balance sensitivity and specificity to accommodate varying clinical needs, such as clinical screening and auxiliary diagnosis, thereby demonstrating greater clinical practicality than single-protein or single-cytokine detection methods.

### Strengths and limitations

#### Strengths

The primary strength of this study lies in its rigorous application of systematic review and meta-analysis methods, strict adherence to the PRISMA guidelines, and prospective registration of the study protocol in PROSPERO, which ensured the standardization and reproducibility of the research process. Strict inclusion and exclusion criteria were applied during the study selection process to ensure the accuracy and completeness of the included literature. A thorough search was conducted across several major databases, including PubMed, Web of Science, and Embase. For quality assessment, the QUADAS-2 tool was used to evaluate the risk of bias and applicability of the included studies. In addition, multivariate meta-regression, subgroup analysis, and sensitivity analysis were employed to explore sources of heterogeneity and verify the robustness of the findings, thereby enhancing the reliability of the study conclusions.

This study also systematically analyzed the diagnostic performance of machine learning and deep learning models for endometriosis, identified key factors influencing model performance, and provided an important evidence-based foundation for developing and optimizing subsequent artificial intelligence diagnostic models for endometriosis. The clinical value analysis of these models provides a clear reference for the clinical application of artificial intelligence technology in endometriosis screening and diagnosis, with significant implications for reducing diagnostic delays in endometriosis and optimizing clinical management strategies.

### Limitations

First, subgroup sample sizes in some analyses were relatively small, which may have reduced statistical power and compromised the reliability of certain subgroup analysis results. Second, although QUADAS-2 was used to assess study quality, this tool may not fully capture methodological challenges unique to artificial intelligence-based diagnostic research, including overfitting, feature selection bias and data leakage. Future investigations should adopt AI-specific appraisal frameworks such as PROBAST or TRIPOD-AI for quality evaluation. Third, although we performed subgroup analyses and meta-regression to explore heterogeneity, the pooling of studies with diverse input data and clinical targets may limit the clinical defensibility of a single summary estimate. The results should therefore be viewed as providing a general indication of diagnostic promise, with modality-specific or lesion-specific inferences requiring further dedicated meta-analyses as the literature grows. Fourth, Deeks' funnel plot asymmetry test suggested the presence of publication bias. While sensitivity analyses verified the robustness of our main results, such bias may still have slightly overestimated the diagnostic performance of the evaluated model. Fifth, and importantly, no included study employed explainable AI (XAI) methods. XAI techniques—such as SHAP, LIME, Grad-CAM, and attention visualization—are essential for translating “black-box” models into clinically trustworthy tools by enabling clinicians to understand which features drive predictions. The absence of XAI in the current literature represents a critical evidence gap, particularly because model interpretability is a prerequisite for clinician trust, bias detection, and safe clinical implementation. Future studies should prioritize the integration of XAI frameworks into model development.

## Conclusion

This study confirms that machine learning and deep learning models demonstrate high overall diagnostic potential for endometriosis. However, given the substantial heterogeneity and methodological limitations of the current evidence, the clinical applicability of these models should be viewed as a promising auxiliary tool for screening and clinical decision support rather than a validated replacement for standard diagnostic pathways.

Although artificial intelligence technology shows considerable promise for endometriosis diagnosis, the relevant research remains in its early stages, with persistent limitations, including small sample sizes, high study heterogeneity, and insufficient prospective studies. Future research should prioritize large-sample, multicenter prospective cohort studies with standardized patient inclusion criteria, gold-standard implementation protocols, and model training and validation strategies to reduce methodological heterogeneity across studies. During model development, attention should be given to balancing sensitivity and specificity, and multimodal fusion models that integrate clinical data, imaging data, and laboratory indices should be constructed to enhance generalizability and diagnostic efficacy. Notably, future large-scale, multicenter prospective studies with rigorous external validation and transparent reporting following standardized guidelines are urgently required to translate the promising diagnostic potential of AI into reliable and feasible clinical practice.

In addition, greater emphasis should be placed on the clinical transformation and validation of artificial intelligence models, including evaluating their practical clinical value in reducing diagnostic delays and optimizing medical resource utilization, and on establishing mechanisms for model updates and maintenance to accommodate dynamic changes in clinical data. ML and DL are expected to play an increasingly significant role in the early identification and precise management of endometriosis, ultimately improving patients' clinical outcomes and quality of life as research designs continue to be refined and artificial intelligence advances.

## Data Availability

The original contributions presented in the study are included in the article/Supplementary Material, further inquiries can be directed to the corresponding author.
