# Supplementary material for: Machine learning and deep learning for diagnosis of endometriosis: systematic review and meta-analysis
Source: Front Med (Lausanne). 2026 Oct 15;13:1907483. doi: 10.3389/fmed.2026.1907483 (PMC13716802; doi:10.3389/fmed.2026.1907483)
Supplement: Supplementary file 1 [file Table1.docx]

**Table S1**

**1.Pubmed**

| Search number | Query | Results |
| --- | --- | --- |
| #1 | "Endometriosis"[Mesh] | 28,236 |
| #2 | Endometrioses"[Title/Abstract] OR "Endometrioma"[Title/Abstract] OR "Endometriomas"[Title/Abstract] OR "Chocolate Cyst"[Title/Abstract] OR "Pelvic Endometriosis"[Title/Abstract] OR "Deep Infiltrating Endometriosis"[Title/Abstract] OR "Deep Endometriosis"[Title/Abstract] OR "Rectovaginal Endometriosis"[Title/Abstract] OR "Ovarian endometriosis"[Title/Abstract] OR "Peritoneal endometriosis"[Title/Abstract] OR "Extra-pelvic endometriosis"[Title/Abstract] OR "Bowel endometriosis"[Title/Abstract] OR "Bladder endometriosis"[Title/Abstract] OR "Ureteral endometriosis"[Title/Abstract] OR "EMS"[Title/Abstract] | 84,489 |
| #3 | #1OR#2 | 48338 |
| #4 | "Machine Learning"[Mesh] | 107,477 |
| #5 | "Deep Learning"[Title/Abstract] OR "Artificial Intelligence"[Title/Abstract] OR "Prediction Model"[Title/Abstract] OR "residual network"[Title/Abstract] OR "Neural Network"[Title/Abstract] OR "convolution"[Title/Abstract] OR "Intelligent System"[Title/Abstract] OR "Statistical Learning"[Title/Abstract] OR "Deep Learning Network"[Title/Abstract] OR "random forest"[Title/Abstract] OR "computational intelligence"[Title/Abstract] OR "machine intelligence"[Title/Abstract] OR "computer reasoning"[Title/Abstract] OR "computer vision"[Title/Abstract] OR "decision tree"[Title/Abstract] OR "naive bayes"[Title/Abstract] OR "support vector machine"[Title/Abstract] OR "support vector network"[Title/Abstract] OR "natural language process"[Title/Abstract] OR "unsupervised learning"[Title/Abstract] OR "supervised learning"[Title/Abstract] ) | 354,763 |
| #6 | 4OR5 | 390,684 |
| #7 | "Area Under Curve"[Mesh] | 47,755 |
| #8 | "AUC"[Title/Abstract] OR "C-statistic"[Title/Abstract] OR "area under the receiver operator curve"[Title/Abstract] OR "Sensitivity"[Title/Abstract] OR "Specificity"[Title/Abstract] OR "Positive Predictive Value"[Title/Abstract] OR "Precision"[Title/Abstract] OR "Accuracy"[Title/Abstract] OR "F1-score"[Title/Abstract] curve[Title/Abstract]OR(Sensitivity[Title/Abstract]OR(Specificity[Title/Abstract]OR(Positive Predictive Value[Title/Abstract]OR(Precision[Title/Abstract]OR(Accuracy[Title/Abstract]OR(F1-score[Title/Abstract] | 2,286,441 |
| #9 | 7OR8 | 2,306,532 |
| #10 | #3 AND #6 AND #9 | 190 |

**2.Cochrane**

| Search number | Query | Results |
| --- | --- | --- |
| #1 | MeSH descriptor: [Endometriosis] explode all trees | 1317 |
| #2 | (Endometrioses):ti,ab,kw OR (Endometrioma):ti,ab,kw OR (Endometriomas):ti,ab,kw OR ("Chocolate Cyst"):ti,ab,kw OR ("Pelvic Endometriosis"):ti,ab,kw OR ("Deep Infiltrating Endometriosis"):ti,ab,kw OR ("Deep Endometriosis"):ti,ab,kw OR ("Rectovaginal Endometriosis"):ti,ab,kw OR ("Ovarian endometriosis"):ti,ab,kw OR ("Peritoneal endometriosis"):ti,ab,kw OR ("Extra-pelvic endometriosis"):ti,ab,kw OR ("Bowel endometriosis"):ti,ab,kw OR ("Bladder endometriosis"):ti,ab,kw OR ("Ureteral endometriosis"):ti,ab,kw OR (EMS):ti,ab,kw | 2197 |
| #3 | #1 OR #2 | 3183 |
| #4 | MeSH descriptor: [Machine Learning] explode all trees | 1222 |
| #5 | ("Deep Learning"):ti,ab,kw OR ("Artificial Intelligence"):ti,ab,kw OR ("Prediction Model"):ti,ab,kw OR ("residual network"):ti,ab,kw OR ("Neural Network"):ti,ab,kw OR ("convolution"):ti,ab,kw OR ("Intelligent System"):ti,ab,kw OR ("Statistical Learning"):ti,ab,kw OR ("Deep Learning Network"):ti,ab,kw OR ("random forest"):ti,ab,kw OR ("computational intelligence"):ti,ab,kw OR ("machine intelligence"):ti,ab,kw OR ("computer reasoning"):ti,ab,kw OR ("computer vision"):ti,ab,kw OR ("decision tree"):ti,ab,kw OR ("naive bayes"):ti,ab,kw OR ("support vector machine"):ti,ab,kw OR ("support vector network"):ti,ab,kw OR ("natural language process"):ti,ab,kw OR ("unsupervised learning"):ti,ab,kw OR ("supervised learning"):ti,ab,kw | 9135 |
| #6 | #4 OR #5 | 9548 |
| #7 | MeSH descriptor: [Area Under Curve] explode all trees | 8979 |
| #8 | ("Deep Learning"):ti,ab,kw OR ("Artificial Intelligence"):ti,ab,kw OR ("Prediction Model"):ti,ab,kw OR ("residual network"):ti,ab,kw OR ("Neural Network"):ti,ab,kw OR ("convolution"):ti,ab,kw OR ("Intelligent System"):ti,ab,kw OR ("Statistical Learning"):ti,ab,kw OR ("Deep Learning Network"):ti,ab,kw OR ("random forest"):ti,ab,kw OR ("computational intelligence"):ti,ab,kw OR ("machine intelligence"):ti,ab,kw OR ("computer reasoning"):ti,ab,kw OR ("computer vision"):ti,ab,kw OR ("decision tree"):ti,ab,kw OR ("naive bayes"):ti,ab,kw OR ("support vector machine"):ti,ab,kw OR ("support vector network"):ti,ab,kw OR ("natural language process"):ti,ab,kw OR ("unsupervised learning"):ti,ab,kw OR ("supervised learning"):ti,ab,kw | 13493 |
| #9 | #7 OR #8 | 139399 |
| #10 | #3 AND #6 AND #9 | 7 |

**3.Embase**

| Search number | Query | Results |
| --- | --- | --- |
| #1 | 'endometriosis'/exp | 58816 |
| #2 | (Endometrioses OR Endometrioma OR Endometriomas OR "Chocolate Cyst" OR "Pelvic Endometriosis" OR "Deep Infiltrating Endometriosis" OR "Deep Endometriosis" OR "Rectovaginal Endometriosis" OR "Ovarian endometriosis" OR "Peritoneal endometriosis" OR "Extra-pelvic endometriosis" OR "Bowel endometriosis" OR "Bladder endometriosis" OR "Ureteral endometriosis" OR EMS):ti,ab | 39434 |
| #3 | #1 OR #2 | 86,769 |
| #4 | 'machine learning'/exp | 665,383 |
| #5 | 'machine learning':ti,ab OR 'deep learning':ti,ab OR 'artificial intelligence':ti,ab OR 'prediction model':ti,ab OR 'residual network':ti,ab OR 'neural network':ti,ab OR convolution:ti,ab OR 'intelligent system':ti,ab OR 'statistical learning':ti,ab OR 'deep learning network':ti,ab OR 'random forest':ti,ab OR 'computational intelligence':ti,ab OR 'machine intelligence':ti,ab OR 'computer reasoning':ti,ab OR 'computer vision':ti,ab OR 'decision tree':ti,ab OR 'naive bayes':ti,ab OR 'support vector machine':ti,ab OR 'support vector network':ti,ab OR 'natural language process':ti,ab OR 'unsupervised learning':ti,ab OR 'supervised learning':ti,ab | 492,765 |
| #6 | #4 OR #5 | 830,741 |
| #7 | 'area under the curve '/exp | 254,618 |
| #8 | "Area Under Curve":ti,ab OR AUC:ti,ab OR "C-statistic":ti,ab OR "area under the receiver operator curve":ti,ab OR Sensitivity:ti,ab OR Specificity:ti,ab OR "Positive Predictive Value":ti,ab OR Precision:ti,ab OR Accuracy:ti,ab OR "F1-score":ti,ab | 2,943,806 |
| #9 | #7 OR #8 | 3,042,241 |
| #10 | #3 AND #6 AND #9 | 524 |

**4.Web of science核心库**

| Search number | Query | Results |
| --- | --- | --- |
| #1 | TS=("Endometrioses" OR "Endometrioma" OR "Endometriomas" OR "Chocolate Cyst" OR "Pelvic Endometriosis" OR "Deep Infiltrating Endometriosis" OR "Deep Endometriosis" OR "Rectovaginal Endometriosis" OR "Ovarian endometriosis" OR "Peritoneal endometriosis" OR "Extra-pelvic endometriosis" OR "Bowel endometriosis" OR "Bladder endometriosis" OR "Ureteral endometriosis" OR "EMS") | 41366 |
| #2 | TS=("Machine Learning" OR "Deep Learning" OR "Artificial Intelligence" OR "Prediction Model" OR "residual network" OR "Neural Network" OR "convolution" OR "Intelligent System" OR "Statistical Learning" OR "Deep Learning Network" OR "random forest" OR "computational intelligence" OR "machine intelligence" OR "computer reasoning" OR "computer vision" OR "decision tree" OR "naive bayes" OR "support vector machine" OR "support vector network" OR "natural language process" OR "unsupervised learning" OR "supervised learning") | 1828574 |
| #3 | TS=("Area Under Curve" OR "AUC" OR "C-statistic" OR "area under the receiver operator curve" OR "Sensitivity" OR "Specificity" OR "Positive Predictive Value" OR "Precision" OR "Accuracy" OR "F1-score") | 5142559 |
| #4 | #1 AND #2 AND #3 | 432 |

5.SCOPUS

| Search number | Query | Results |
| --- | --- | --- |
| #1 | Title-Abs-Key("Endometriosis" or "Endometrioses" or "Endometrioma" or "Endometriomas" or "Chocolate Cyst" or "Pelvic Endometriosis" or "Deep Infiltrating Endometriosis" or "Deep Endometriosis" or "Rectovaginal Endometriosis" or "Ovarian endometriosis" or "Peritoneal endometriosis" or "Extra-pelvic endometriosis" or "Bowel endometriosis" or "Bladder endometriosis" or "Ureteral endometriosis" or "EMS") | 97870 |
| #2 | Title-Abs-Key("Machine Learning" or "Deep Learning" or "Artificial Intelligence" or "Prediction Model" or "residual network" or "Neural Network" or "convolution" or "Intelligent System" or "Statistical Learning" or "Deep Learning Network" or "random forest" or "computational intelligence" or "machine intelligence" or "computer reasoning" or "computer vision" or "decision tree" or "naive bayes" or "support vector machine" or "support vector network" or "natural language process" or "unsupervised learning" or "supervised learning") | 3,521,303 |
| #3 | Title-Abs-Key("Area Under Curve" or "AUC" or "C-statistic" or "area under the receiver operator curve" or "Sensitivity" or "Specificity" or "Positive Predictive Value" or "Precision" or "Accuracy" or "F1-score") | 7,846,788 |
| #4 | #1 AND #2 AND #3 | 897 |
